# Advanced Therapies for Human Immunodeficiency Virus

**DOI:** 10.3390/medsci12030033

**Published:** 2024-07-18

**Authors:** Daniel Josef Lindegger

**Affiliations:** 1 Independent Researcher, 6000 Lucerne, Switzerland; lindeggerdaniel@gmail.com; 2 Independent Researcher, London SW1A2JR, UK

**Keywords:** advanced therapy medicinal products (ATMP), cell therapy, gene therapy, human immunodeficiency virus (HIV), trials, vaccines

## Abstract

Human Immunodeficiency Virus (HIV) remains a significant global health challenge with approximately 38 million people currently having the virus worldwide. Despite advances in treatment development, the virus persists in the human population and still leads to new infections. The virus has a powerful ability to mutate and hide from the human immune system in reservoirs of the body. Current standard treatment with antiretroviral therapy effectively controls viral replication but requires lifelong adherence and does not eradicate the virus. This review explores the potential of Advanced Therapy Medicinal Products as novel therapeutic approaches to HIV, including cell therapy, immunisation strategies and gene therapy. Cell therapy, particularly chimeric antigen receptor T cell therapy, shows promise in preclinical studies for targeting and eliminating HIV-infected cells. Immunisation therapies, such as broadly neutralising antibodies are being investigated to control viral replication and reduce reservoirs. Despite setbacks in recent trials, vaccines remain a promising avenue for HIV therapy development. Gene therapy using technologies like CRISPR/Cas9 aims to modify cells to resist HIV infection or eliminate infected cells. Challenges such as off-target effects, delivery efficiency and ethical considerations persist in gene therapy for HIV. Future directions require further research to assess the safety and efficacy of emerging therapies in clinical trials. Combined approaches may be necessary to achieve complete elimination of the HIV reservoir. Overall, advanced therapies offer new hope for advancing HIV treatment and moving closer to a cure.

## 1. Introduction

### 1.1. The Origins of HIV and the Global Pandemic

Human Immunodeficiency Virus (HIV), discovered in the early 1980s, is a retrovirus of the genus Lentivirus which affects human T-cells [1]. The retrovirus is capable of converting its RNA genome into DNA through the process of reverse transcription. The DNA is integrated into the host cell’s genome allowing the virus to replicate and persist within the host cell. The infection which primarily targets CD4+ T-cells is chronic and hallmarked by a progressive destruction of Lymphocytes. When the immune system becomes dysfunctional due to low T-cell count, Acquired Immunodeficiency Syndrome (AIDS) can manifest as a syndrome with distinct symptoms [2].

There are two main types of HIV, type 1 (HIV-1) and type 2 (HIV-2). HIV-1 is the most common and virulent strain, responsible for most of the HIV infections worldwide. HIV-2 is less common and is primarily found in West Africa, although it shares many similarities with HIV-1 in terms of its structure and mode of transmission. HIV has to be seen as a complex retrovirus that has evolved and developed many mechanisms to evade the immune system and persist in the human body in reservoirs. Globally, there is still a significant disease burden with approximately 38 million people with the virus [3].

### 1.2. HIV’s Mechanism of Action

HIV undergoes several key steps when entering and surviving in a host organism. Initially, the host cell interacts with glycoprotein gp120 of the viral envelope via the CD4 receptor. This interaction allows viral attachment and fusion and subsequent entry of the virus into the host cell. This process is accompanied by the release of viral RNA and different enzymes including reverse transcriptase, integrase and protease into the host cell’s cytoplasm. Reverse transcriptase converts viral RNA genome into double-stranded DNA which allows the integration of the viral DNA into the host genome. This integration is an important step for the virus to evade the human immune system. The integrated provirus will then undergo transcription and translation to generate viral RNA and proteins which are released from the cell through budding. The function of the host cell is progressively impaired which leads to immune cell destruction. Once the immune system of the host is sufficiently compromised, acquired immunodeficiency syndrome (AIDS) manifests, which is characterised by opportunistic infections and cancer susceptibility [4,5].

### 1.3. Possibility of a Cure?

In 2009, a case report of a patient positive for HIV with acute myeloid leukaemia who underwent a hematopoietic stem cell transplant from a donor homozygous for the C-C chemokine receptor type 5 (CCR5)-delta32 mutation was published. The CCR5 receptor is a seven-transmembrane, G-protein-coupled receptor which regulates the trafficking and effector functions of memory/effector T-Lymphocytes, macrophages and immature dendritic cells. While mutations in the receptor lead to a shorter life expectancy for an individual, the alteration confers enhanced resistance to or immunity against HIV infection due to the impaired binding, entry and spread of the virus in the presence of a mutation [6]. The patient, also called the “Berlin patient”, who received the stem cell transplant, was subsequently free from viral rebound for 20 months after transplantation and discontinuation of the antiretroviral therapy could be achieved. The treatment approach was validated in a second patient, called “the London” patient, who was free from disease for 30 months and thus was considered to be cured [7]. The outcomes demonstrate the important role of CCR5 in the maintenance of HIV-1 infection [8] and the possibility of a cure for HIV. 

### 1.4. Enduring Hurdles in Therapeutic Discovery

There are different bio-medical challenges to finding a cure for HIV. HIV-infected cells are not very abundant and are located in anatomical sites which are poorly accessible. The mechanisms of HIV latency and reactivation are not fully understood, especially in the context of virus diversity and viral escape phenomena. Viral diversity describes the extensive genetic variations of the HI Virus which is primarily driven by its high mutation rate, short replication cycle and the ability of the virus to recombine its genetic material [9]. Viral escape is a strategy of the virus to avoid degradation by the host immune system through rapid genetic mutations. Specifically, alterations to the viral surface proteins as a consequence of genetic mutation enable the virus to become less recognisable by antibodies or lymphocytes and therefore allow the virus to evade the host’s immune response or a therapeutic mechanism of action [10]. 

After successful immune system evasion due to alteration of the virus’ geno- or phenotype, HIV can remain dormant within infected cells, primarily in long-lived resting memory CD4+ T-cells, which characteristically are devoid of nuclear forms of key host transcription factors such as NFκB and NFAT. When persisting over years in these cells, the virus alters the host cell’s genome. While in this state, the virus generally does not replicate despite remaining replication competent. Clinically, HIV latency explains the low but persistent levels of viraemia in patients under retroviral treatment [11,12].

HIV’s genetic variability and capacity to alter its characteristics and reside in an intracellularly dormant state increase the difficulty of developing a treatment or vaccine and make monitoring of the therapeutic efficacy necessary [13]. The development of a cure will require a more complex strategy which addresses all challenges that the virus imposes on the host organism.

### 1.5. Current Treatment Modalities

The currently available standard treatment is Antiretroviral Therapy (ART), which aims to suppress viral replication, restore immune function and prevent disease progression. The recommended initial treatment scheme for ART-naïve individuals is a combination of two nucleoside reverse transcriptase inhibitors (NRTIs) with an integrase strand transfer inhibitor (INSTI) according to recent national guidelines from the United States of America [14]. While ART can effectively control HIV replication, it does not eradicate the virus from the body and lifelong treatment is required to maintain viral suppression [15]. ART is generally well tolerated by the patients but complications such as long-term toxicity, drug resistance or adherence problems remain [16]. In addition, ART has a dual effect on the immune system: ART diminishes the autoimmune response associated with chronic HIV infections, which manifests as immune activation, chronic inflammation and subsequent tissue damage. Conversely, ART can induce or exacerbate preexisting autoimmune conditions in the context of immune reconstitution which may lead to autoimmune thyroiditis, autoimmune hepatitis, systemic sclerosis or lupus-like syndromes [17,18]. Therefore, there is an important need for novel therapeutic approaches with curative potential or at least sustained viral suppression or immune reconstitution.

Latency-reversing agents (LRAs) are a class of drugs which reactivate latent HIV within CD4 cells allowing ART and the body’s immune system to attack the virus. The class consisting of histone deacetylase inhibitors, protein kinase C agonists or Toll-like receptor agonists are currently under investigation by the Food and Drug Administration (FDA) and are pending approval [19]. 

The currently available treatments, prescribed mostly as a combination of different pharmaceuticals, manage to effectively suppress the HIV load, often to an undetectable level. The treatment has also converted HIV/AIDS from a fatal to a manageable chronic condition with an extended to normal lifespan and an improved quality of life. However, there are still unfavourable disease courses and complications of disease and treatment such as toxicity or allergic reactions. In addition, there are medical adherence issues related to the life-long treatment and inequality of the accessibility of the medications persists due to their cost and limited availability. Drug-resistance problems, as a consequence of the adaptability and versatility of the virus, increase the need to develop new treatments. Finding a cure that permanently eliminates the latent HIV reservoir (which can lead to rebound or further infection) would remove all described limitations and issues related to the current treatment that can only control the virus. 

### 1.6. Emerging ATMPs Offering Hope

Advanced Therapy Medicinal Products (ATMPs) represent a novel class of therapies which use complex cell-therapeutic or molecular methods such as stem cell modification, gene therapy or modification and vaccines. These complex therapies are required if conventional treatments are unavailable or not sufficiently effective [20]. ATMPs represent an important opportunity for HIV treatment because the condition is still not completely curable and has a large disease burden, especially in disadvantaged populations. In recent years, significant progress has been made in the development of ATMPs for HIV. This review provides an in-depth analysis of the latest evidence on the use of ATMPs for HIV, including their mechanism of action, clinical efficacy, challenges and prospects (Table 1). This review aims to depict the current landscape of advanced therapeutics to provide an overview and simultaneously serve as a foundation for the development of further therapeutic concepts.

## 2. Cell Therapy against HIV

### 2.1. Chimeric Antigen Receptor (CAR) T Cell Therapies

Cell therapeutic concepts make use of genetically modified immune cells with the purpose of improving the robustness of the immune system and controlling HIV replication. One of the most studied cell-based therapies for HIV is chimeric antigen receptor (CAR) T-cell therapy which targets HIV antigens [51]. The treatment has been used successfully in many haematologic malignancies with good results [52]. CAR T cells lead to reductions in viral load and immune reconstitution and have shown strong antiviral activity in preclinical models [53]. Establishing long-term antiviral control, however, remains a limitation that needs to be addressed due to the lack of persistence of CAR T cell activity. 

CAR-T therapy involves genetically modifying a patient’s T cells to target cells expressing disease epitopes, potentially leading to clearance of HIV-infected cells. The aim of CAR T cell therapy is to generate immunogenic memory and surveillance to eliminate HIV infection in its reservoirs. It is important to develop a treatment which does not lead to graft-versus-host disease, a complication which could occur due to the autologous nature of the approach. Animal studies have shown promising results for genetically engineered autologous T-cells, with CAR-T cells persisting for over 2 years without adverse effects and effectively targeting HIV-infected cells [54,55]. While in vitro and animal model studies have demonstrated viral clearance, clinical trials for HIV-1 CAR-T therapy are limited, necessitating further investigation.

### 2.2. Haematopoietic Stem Cell Transplantation (HSCT)

Haematopoietic stem cell transplantation (HSCT) has been explored as a potential functional HIV cure since the 1980s, with cases like the ‘London patient’ and ‘Berlin patient’ showing promise. HSCT aims to eliminate viral reservoirs by replacing the patient’s immune system with donor stem cells, some of which lack the CCR5 receptor. Despite successes, HSCT faces challenges such as high treatment risks, strict patient and donor criteria and the rarity of suitable donors. While some cases show potential for HIV cure, widespread adoption of HSCT for HIV treatment requires overcoming significant hurdles [56].

### 2.3. Limitations and Challenges of Cell Therapies

Cell therapies have several limitations in the context of HIV, especially in the clinical setting. Safety concerns such as graft-versus-host-disease (GVHD) and cytokine release syndrome (CRS) can lead to relevant complications in the context of allogeneic stem cell transplantation. The stability of the transplanted cells in the body is critical for the effectiveness of the treatment; however, achieving persistent engrafting is not guaranteed. This is especially important in the context of latent HIV reservoirs, which are hidden in tissues which are difficult to access. In addition, cell therapies are not robust against the mutation of the virus which allows an “immune escape” from the designed cell therapies. Besides the clinical challenges, the production process requiring advanced laboratory facilities and expertise imposes barriers to accessibility, scalability and standardisation of the treatment. Resulting ethical and regulatory constraints increase the costs of manufacturing and supplying the treatment [28].

### 2.4. Emerging Alternative Cell-Based Therapies

Other cell-based treatments besides CAR T and HSCT are under investigation. These include Natural Killer (NK) cell therapies whose function can be enhanced with genetic modification [25]. A similar concept is the use of chimeric antigen receptor macrophages (CAR-M), which are genetically engineered macrophages with enhanced ability to target and destroy HIV cells in reservoirs [26]. Different types of modified T-cell therapies are in development which aim to modify the immune cells genetically to make them more resistant to the virus (such as CCR5-gene disruption, [27]) or involve modification of the T-cell receptor (TCR) which can then detect and eliminate HIV-infected cells with a high specificity [28]. Further approaches focus on the modification of regulatory T cells (Treg) to control HIV replication and reducing latent reservoirs [29]. Genetically edited induced pluripotent stem cells (iPSC) derived from HIV-infected patients can be used to give rise to immune cells that are resistant to HIV [30].

## 3. Immunisation Therapies

### 3.1. Antibodies against HIV

Latently HIV-infected cells which do not demonstrate active virus replication cannot be targeted by the host immune system because no viral proteins are expressed on the cell surface. These cells are however capable of reinfecting the host after ART is discontinued. Antibodies could therefore be used to eliminate these virus reservoirs. Broadly neutralising antibodies (bNAbs) are an approach to reducing viremia by targeting CD4 and HIV-specific epitopes using viral isolates from HIV-infected donor blood [57].

Data from animal models and early-phase clinical trials demonstrate that bNAbs have antiviral activity and delay viral rebound after ART interruption. These studies demonstrate that bNAbs not only supress plasma viral RNA but also reduce proviral DNA in various tissues, indicating the potential clearance of infected cells through Fc-mediated mechanisms. Clinical data from the first in-human trials have confirmed the ability of single administrations of bNAbs or bNAb combinations to significantly reduce viral loads in individuals infected with HIV-1. Antibodies with single epitopes have a small immune spectrum and short half-life in the human body; thus, long-term protection and viral suppression are not achieved. The degree of viral control and the emergence of antibody-resistant viruses vary among studies, highlighting the need for further investigation into the size of the inducible viral reservoir and the development of strategies to enhance antibody efficacy [58,59,60]. Combined immunisation with multiple epitope antibodies can therefore be a concept that can reduce HI viral load more effectively. BNAbs VRC01 and 3BNC117, which target CD4+ T lymphocytes, have completed phase 1 trials [61]. Ibalizumab, another CD4+ specific antibody, has completed a phase III trial which has demonstrated that the antibody can effectively block the HIV 1 binding site [62]. UB-421 underwent a phase II clinical study which demonstrated that the antibody treatment led to stable CD4+ cells and viremia in the study subjects [63]. 

#### 3.1.1. Emerging Alternative Antibody-Mediated Therapeutics

Further strategies for antibody-mediated therapeutics involve antibody-dependent cytotoxicity (ADCC) and antibody-dependent phagocytosis (ADCP). Enhanced ADCC activity has been observed in serum samples from patients with HIV and has been associated with protection against infection in vaccine studies [64]. Phagocytic activity mediated by Fcγ receptors on immune cells offers another mechanism for clearing HIV virions and infected cells. Fc-engineered antibodies with optimised Fc domains for enhanced effector functions show promise in improving antibody efficacy and reducing the risk of viral escape [65]. 

#### 3.1.2. Limitations and Challenges of Antibody-Based Therapies

The viral diversity in the latent reservoir remains a major obstacle for antibody-based curative approaches. ART cessation leads to a rebound of diverse viral populations from multiple tissue sources, including the central nervous system (CNS), which complicates efforts to eradicate the reservoir. Identifying viral resistance in patients before antibody treatment and selecting bNAb combinations with a wide viral coverage are crucial steps in designing effective antibody regimens for HIV reservoir eradication strategies. Bioinformatic tools such as computational models may guide the creation of proviral sequences to predict bNAb susceptibility to develop a personalised antibody therapy [66]. 

Further obstacles to the development of immunisation therapies are the heterogeneity of HIV strains which require the development of broad-spectrum therapies capable of targeting multiple HIV variants at the price of specificity. The high mutation rate of the HI Virus is a further challenge for immunisation therapies due to the development of resistance. This resistance may limit the effectiveness of treatments and reduce the durability of the body’s immune response, making treatment monitoring and booster treatments a necessity. Autoimmune reactions or excessive immune responses can occur as a consequence of immunisation treatments and lead to tissue damage or inflammation. The complexity of immunisation treatments and their costs require substantial funding sources and regulatory support [67]. 

While antibodies hold promise as advanced therapeutic agents in the treatment of HIV, further research is required to enhance their efficacy, address viral diversity and develop personalised treatment approaches for achieving long-term viral suppression and possibly a cure. Personalised treatments have to be a focus for this treatment group as immunisation therapies have to take account of an individual’s genetic background, differences in the immune system and the specific HIV strain. Personalised biomarkers are needed to predict the response and effectiveness of immune treatments and reduce adverse effects. 

### 3.2. Vaccines against HIV

Therapeutic vaccines aim to stimulate HIV-specific immune responses to control viral replication. Several therapeutic vaccine candidates have been evaluated in clinical trials. Common subclasses are peptide-based vaccines, viral vectors and dendritic cell vaccines [68]. Early-phase trials have demonstrated some effectiveness in improving immune response against the virus; however, a sustained therapeutic effect has not been achieved yet [69]. Vaccine design is challenging due to immune escape and viral diversity as well as host-specific factors. Specifically, the high mutation and recombination rate of the Env protein during replication increase the difficulty of designing a functioning vaccine substantially. 

#### 3.2.1. HIV Vaccine Trials

The RV144 trial in Thailand tested a vaccine regimen called ALVAC-HIV and AIDSVAX [70]. The vaccine trials demonstrated a partial level of protection against HIV infection but were not considered sufficiently effective. However, the trial provided valuable insights into the interaction between the vaccine and the immune system, inspiring many subsequent vaccine developments. 

Currently, different vaccine candidates are under investigation, such as viral vector-based vaccines, protein subunit vaccines, DNA vaccines and mosaic vaccines. These candidates aim to induce a broad and potent immune response against HIV. Unfortunately, over the last year, many trials have been discontinued because the vaccine product could not demonstrate sufficient effectiveness. The Mosaico (HVTN 706/HPX3002) phase 3 trial was one of the promising candidates which was stopped after a planned interim analysis because there was no evidence that the vaccine was efficacious in preventing HIV [71,72]. The companion trial, called Imbokodo (HVTH 705/HPX2008) was stopped due to low efficacy in August 2021 [73]. The vaccines failed to elicit antibodies capable of neutralising the highly diverse HIV strains. Additionally, a sustained immune response could not be induced by the vaccines [74]. The Merck Ad5/HIV, a non-replicating adenovirus-based vaccine that had achieved promising results in rhesus macaques, failed in further trials due to having a cytotoxic capacity that was inferior to expectations [75,76].

Common reasons for the failure to develop broad and effective HIV vaccines are rooted in the high genetic variability and antigen variation of the virus. In particular, the alterations of surface glycoproteins gp120 and gp41 can impede recognition by the immune system. An adequate activation of the humoral and cellular components of the immune system is required to have an adequate and long-lasting response. Latent reservoirs and the immune evasion mechanism pose additional challenges for vaccine development [77,78].

While there is ongoing interest in therapeutic vaccines which could help control viral replication in individuals already infected with HIV (such as the IAVI G003 HVTN302 trial), an important focus of HIV vaccine research is currently on the development of vaccines that prevent HIV infection, such as CAPRISA 12B [71].

#### 3.2.2. mRNA Vaccines—A Development Spurred by the COVID Pandemic

The COVID-19 pandemic has led to the development of mRNA platforms which are used in many of the current HIV studies. There are several hurdles to overcome before an mRNA HIV vaccine is possible. The detailed mechanism of immunogenicity in mRNA has to be elucidated including clarification of which cell types are implicated in the immune response and how mRNA encoded immunogens interact with different immune cell types [79].

Despite the challenges, mRNA-based vaccine strategies are considered highly promising for future successful immunologic treatment. Zhang et al. used molecules which code for HIV-specific structures (envelop glycoprotein, Env) and combined them with other structural HIV-specific proteins (Gag) in lipid nanoparticles to form virus-like particles (VLPs) in vivo. The Env-expressing VLPs induced bNAbs that had an antiviral effect in a macaque model [80].

A report by Schiffner et al. details an animal experiment in which a precursor B cell was able to produce multiple types of bNAbs (class 10E8) which target glycoprotein gp41, a surface molecule involved with the entry into human cells. GP41 is located in a recessed crevice on the surface of the virus and has previously not been reached with vaccine immunogens. Mice and macaque monkeys were vaccinated with engineered nanoparticles equipped with immunogens mimicking a specific part of gp41. The immunogens managed to elicit responses from 10E8 B cell precursors to produce bNAbs, some of which are capable of reaching the GP41 region [81].

## 4. Gene Therapy against HIV

The general strategy for this type of advanced therapeutic is to modify the genetic configuration of cells to make them more resistant to the HI Virus or enhance capabilities to eliminate virus-infected cells. A popular and promising tool includes gene editing systems such as the CRISPR/Cas9 system [82]. A promising target is the CCR5 co-receptor, an entry port for the HI Virus into the CD4+ Lymphocyte [83]. Clinical trials for gene therapies using CCR5 as a target have shown promising results, with some patients achieving long-term viral suppression [42]. Currently, safety and efficacy have to be improved by reducing off-target effects and immune responses to viral vectors. 

### 4.1. Gene-Editing Technologies

Different gene therapeutic strategies have been employed such as the introduction of antiviral genes into the body or the modification of infected cells to confer resistance to HIV. Key receptors such as CCR5 and CXCR4 are frequently targeted. Various gene editing techniques including ribozymes [84], interference RNA (RNAi) [43], zinc-finger nucleases (ZFN) [85], transcriptional activator-like effector nucleases (TALENs) [40] and clustered regularly interspace short palindromic repeats/associated protein 9 (CRISPR/Cas9) are utilised to disrupt the integrated HIV provirus or silence the expression of co-receptors, thereby preventing HIV rebound after treatment interruption [86].

Ribozymes cleave specific RNA sequences and have shown potential in inhibiting HIV replication. While delivery efficiency remains a challenge, clinical trials have demonstrated the safety and efficacy of ribozymes with promising results in reducing viral load and increasing CD4+ T cell count [84]. RNAi inhibits gene expression by introducing small interfering RNA (siRNA) or short hairpin RNA (shRNA) into cells. Various genes within HIV, including LTR, Gag and Vif, can be targeted by RNAi, showing significant inhibitory effects on viral replication. Combining RNAi with other strategies has shown enhanced efficacy against HIV [43].

ZFN and TALENs are engineered endonucleases that target specific DNA sequences, such as CCR5, to disrupt HIV replication. Clinical trials have demonstrated reduced viral DNA levels and delayed viral rebound, although challenges remain regarding off-target effects [40,85]. CRISPR/Cas9 is a versatile gene editing tool with diverse applications in HIV therapy. CRISPR/Cas9 can target viral genes or disrupt integrated provirus, offering potential for HIV cure. Clinical trials have shown promising results in reducing viral DNA levels and achieving HIV resistance in CD4+ T cells [86].

### 4.2. Vector-Based Gene Therapies

There are different advanced therapy concepts available for vector-based gene therapies which hold promise as a potential treatment strategy for HIV/AIDS, with lentiviral vectors being a key focus of current research. Lentiviral vectors, derived from viruses like HIV-1, have the ability to genetically modify nondividing cells, making them particularly attractive for long-term treatment solutions. Despite concerns regarding safety, including insertional mutagenesis and the development of replication-competent lentivirus (RCL), lentiviral vectors offer advantages such as efficient gene delivery and potential anti-HIV effects [46,87].

Efforts to enhance the safety of lentiviral vectors have led to the development of modified vectors with a reduced risk of recombination and RCL formation. These modifications include the use of self-inactivating (SIN) vectors and alterations in the packaging system to minimise the potential for RCL production. Lentiviral vectors are being tested in clinical trials to evaluate their safety and efficacy in humans [88]. 

Another approach to gene therapy for HIV/AIDS involves the use of recombinant SV40-derived vectors. These vectors, although not yet in clinical trials, offer advantages such as high transduction efficiency and long-lasting transgene expression. They have been studied extensively in tissue culture and animal models, demonstrating strong inhibition of HIV-1 replication [89]. 

Various anti-HIV transgenes, including single-chain Fv antibodies, ribozymes, antisense and siRNAs, have been incorporated into SV40-derived vectors, showing promising results for inhibiting HIV-1 in vitro and in vivo. Combination strategies involving multiple transgenes delivered sequentially have demonstrated synergistic protection against HIV-1 [90].

### 4.3. Limitations and Challenges of Gene Therapies

Despite significant progress, gene therapy for HIV has to face different challenges. On a biomedical level, delivery efficiency is a major barrier to gene editing techniques. Off-target effects (including the risk of cancer and germ-line mutations) due to insufficient specificity are a second important concern. The specificity issue is particularly pertinent in the context of HIV due to the virus’ high mutation rate. A single point mutation of the virus is sufficient to make a siRNA therapeutic ineffective. This can be overcome by addressing highly conserved sequences or with the introduction of multiple siRNAs capable of targeting viral escape mutants. For vector-based treatments, insertional mutagenesis and immune reactions to the AAV vectors present significant limitations to research and development success. Continued research is essential to address safety concerns, improve transfection efficiency, and optimise long-term therapeutic outcomes. Various innovative gene therapy methods, including antisense nucleic acids, RNA decoys, and peptide inhibitors, hold promise for future HIV treatment strategies.

## 5. Future Directions and Conclusions

The landscape of HIV treatment is evolving rapidly with ATMPs offering new hope for individuals living with the virus. From immunologic strategies to cell and gene therapies, current research is exploring new avenues to combat HIV infections and find a cure. 

While CAR T cell therapy shows promising results in preclinical studies, further research is required to assess safety and efficacy in clinical trials. Antibody-based therapies such as broadly neutralising antibodies (bNAbs) are under investigation to control HIV replication and reduce the viral reservoir. ADCC and ADCP offer alternative mechanisms for clearing HIV-infected cells. 

Despite the setbacks in recent vaccine trials, preventive or therapeutic vaccination is still considered a highly promising avenue for the discovery of a cure for HIV. Advances in mRNA platforms, spurred by COVID-19 vaccine development, hold promise for future vaccine candidates. Gene editing technologies, particularly CRISPR/Cas9, offer unprecedented opportunities for HIV cure strategies, although barriers in terms of safety and efficacy remain. 

The formation of an HIV reservoir in the human body remains a central limitation for the successful development of ATMPs for HIV. While different strategies focus on reducing the HIV reservoir, more research is required to find a solution. Vaccines and cell and gene therapies have to be further developed to be effective and reliable against the HI Virus. Research should focus on the further refinement of mRNA-based vaccine concepts including VLPs and bNAbs to promote progression to the clinical stage. If no single treatment strategy leads to success, combined approaches (such as BNAbs, gene edition and immune modulation) may be promising for complete elimination of the HIV reservoir. 

A preventative or curative ATMP would profoundly reshape the clinical management of patients with HIV. Lifelong treatment and monitoring could be discontinued or limited. HIV-associated morbidity and mortality would be reduced thereby enhancing the quality of life of patients. A consequential reduction in HIV transmission would decrease healthcare expenditures and foster a beneficial global health outcome. 

## Figures and Tables

**Table 1:** Overview of Advanced Therapy Medicinal Products including prospective costs.

Category	Treatment	Treatment Cost (Estimate)	References
**Cell Therapy**	CAR T Cell Therapy	Variable, approx. USD 373,000–530,000 per treatment	Levine et al., 2017 [21]; June and Sadelain, 2018 [22]
	Hematopoietic Stem Cell Transplantation (HSCT)	USD 100,000–USD 400,000	Ambinder et al., 2020 [23]; Gupta et al., 2019 [24]
	Natural Killer (NK) Cell Therapy	In development, cost TBD	Mikulak et al., 2017 [25]
	Chimeric Antigen Receptor Macrophages (CAR-M)	In development, cost TBD	Li et al., 2023 [26]
	Modified T-Cell Therapy	In development, cost TBD	Li et al., 2022 [27]; Matsui et al., 2023 [28]
	Regulatory T Cell (Treg) Therapy	In development, cost TBD	Kleinman et al., 2018 [29]
	iPSC-derived Immune Cells	In development, cost TBD	Teque et al., 2020 [30]
**Immunisation Therapies**			
- **Antibodies against HIV**	Broadly Neutralizing Antibodies (bnAbs)	USD 10,000–USD 30,000 per year	Scheid et al., 2016 [31]; Caskey et al., 2017 [32]; Haynes et al., 2023 [33]
- **Vaccines against HIV**	Mosaic-based Vaccines	In development, cost TBD	Barouch et al., 2018 [34]
	DNA Vaccines	In development, cost TBD	Ramirez et al., 2013 [35]
	mRNA based Vaccines	In development, cost TBD	Pardi et al., 2018 [36]; Esteban et al., 2021 [37]
**Gene Therapy**			
- **Gene-editing Technologies**	CRISPR/Cas9 Therapy	In development, cost TBD	Liao et al., 2015 [38]; Khalili et al., 2017 [39]
	Transcription activator-like effector nucleases (TALEN)	In development, cost TBD	Benjamin et al., 2016 [40]
	Zinc Finger Nucleases (ZFNs)	In development, cost TBD	Tebas et al., 2014 [41]; Holt et al., 2010 [42]
	Small interfering RNAs (siRNAs)	In development, cost TBD	Bobbin et al., 2015 [43]; Martinez et al., 2009 [44]; Agbosu et al., 2022 [45]
**Vector-based Gene Therapies**	Lentiviral Vectors	In development, cost TBD	Johnson et al., 2021 [46]; Poeschla et al.,1996 [47]
	Adeno-associated Viral (AAV) Vectors	In development, cost TBD	Priddy et al., 2019 [48]
	Gamma-retroviral Vectors	In development, cost TBD	Maetzig et al., 2011 [49]; Kitawi et al., 2024 [50]

## Data Availability

Not applicable.
